# CD147 and downstream ADAMTSs promote the tumorigenicity of Kaposi's sarcoma-associated herpesvirus infected endothelial cells

**DOI:** 10.18632/oncotarget.6584

**Published:** 2015-12-12

**Authors:** Lu Dai, Jimena Trillo-Tinoco, Yihan Chen, Karlie Bonstaff, Luis Del Valle, Chris Parsons, Augusto C. Ochoa, Jovanny Zabaleta, Bryan P. Toole, Zhiqiang Qin

**Affiliations:** ^1^ Research Center for Translational Medicine and Key Laboratory of Arrhythmias, East Hospital, Tongji University School of Medicine, Shanghai 200120, China; ^2^ Departments of Microbiology/Immunology/Parasitology, Louisiana State University Health Sciences Center, Louisiana Cancer Research Center, New Orleans, LA 70112, USA; ^3^ Department of Medicine, Louisiana State University Health Sciences Center, Louisiana Cancer Research Center, New Orleans, LA 70112, USA; ^4^ Department of Pathology, Louisiana State University Health Sciences Center, Louisiana Cancer Research Center, New Orleans, LA 70112, USA; ^5^ Department of Pediatrics, Louisiana State University Health Sciences Center, Louisiana Cancer Research Center, New Orleans, LA 70112, USA; ^6^ Department of Regenerative Medicine and Cell Biology, Medical University of South Carolina and Hollings Cancer Center, Charleston, SC 29425, USA

**Keywords:** KSHV, Kaposi's sarcoma, CD147, microarray

## Abstract

Kaposi's sarcoma-associated herpesvirus (KSHV) is the etiologic agent of several human cancers, including Kaposi's sarcoma (KS), which preferentially arise in immunocompromised patients and lack effective therapeutic options. We have previously shown that KSHV or viral protein LANA up-regulates the glycoprotein CD147, thereby inducing primary endothelial cell invasiveness. In the current study, we identify the global network controlled by CD147 in KSHV-infected endothelial cells using Illumina microarray analysis. Among downstream genes, two specific metalloproteases, ADAMTS1 and 9, are strongly expressed in AIDS-KS tissues and contribute to KSHV-infected endothelial cell invasiveness through up-regulation of IL-6 and VEGF. By using a KS-like nude mouse model, we found that targeting CD147 and downstream ADAMTSs significantly suppressed KSHV-induced tumorigenesis *in vivo*. Taken together, targeting CD147 and associated proteins may represent a promising therapeutic strategy against these KSHV-related malignancies.

## INTRODUCTION

Kaposi sarcoma-associated herpesvirus (KSHV) represents one of major causative agent of cancers arising in immunocompromised patients, including Kaposi's Sarcoma (KS) [1]. Furthermore, despite the reduced incidence of KS in the era of combined Antiretroviral Therapy (cART) for Human Immunodeficiency Virus (HIV) infection, KS still remains the most common Acquired Immunodeficiency Syndrome (AIDS)-associated tumor and a leading cause of morbidity and mortality in this setting [2, 3]. In addition, a longitudinal study conducted among solid organ transplant recipients in United States reported a high prevalence (15%) of KSHV seropositivity in this population [4]. Transplant recipients who develop primary KSHV infection after transplantation have a relatively high probability of developing KSHV-related malignancies, especially KS [5], which is likely associated with the intensity of immunosuppressive treatment post-transplantation [6]. Therefore, KSHV-induced malignancies, in particular KS, still represent a serious threat to immunosuppressed patients due to the lack of effective therapies. In fact, KSHV has now become a model pathogen for virus-induced cancer research. However, many key questions regarding its mechanisms of oncogenesis still remain unanswered, thus hindering identification of rational targets and development of novel therapeutic strategies.

The multifunctional transmembrane protein, CD147, also known as Emmprin or Basigin, induces the expression and secretion of multiple matrix metalloproteinases (MMPs), thereby promoting tumor cell invasion and other malignant behaviors [7–9]. We recently reported that enhancement of invasiveness for primary endothelial cells (the major cellular components of KS), following *de novo* KSHV infection, resulted from up-regulation of CD147 by the KSHV-encoded latency-associated nuclear antigen (LANA) protein [10]. Further study indicated that PI3K/Akt and MAPK activation of vascular endothelial growth factor (VEGF) was required for CD147-mediated endothelial cell invasion [11]. In addition, CD147 and related proteins are also involved in multidrug-resistance of primary effusion lymphoma (PEL), another KSHV-caused malignancy [12]. These data demonstrate the important role of CD147 in KSHV-associated malignancies. However, the global gene profile controlled by CD147 within primary endothelial cells, in particular KSHV-infected cells, remains unknown. It will also be interested to understand the cellular functions of CD147-downstream proteins *in vitro* and *in vivo*, as well as their clinical relevance within AIDS-KS tumor tissues. In the current study, we used Illumina microarray to identify the global network controlled by CD147 within either CD147-overexpressed or KSHV-infected endothelial cells. We also tested the contribution of two CD147-controlled proteins, ADAMTS (A Disintegrin and Metalloprotease with ThromboSpondin motifs) 1 and 9 to KSHV pathogenesis and their clinical correlations in AIDS-KS tissues. Finally, using a KS-like nude mouse model with KSHV long-term-infected, telomerase-immortalized human umbilical vein endothelial (TIVE-LT) cells [13], we assessed the role of CD147 and downstream ADAMTSs in KSHV-related tumorigenesis *in vivo*.

## RESULTS

### Microarray analysis of the CD147 regulatory network in CD147-overexpressed and KSHV-infected endothelial cells

**Figure 1:**
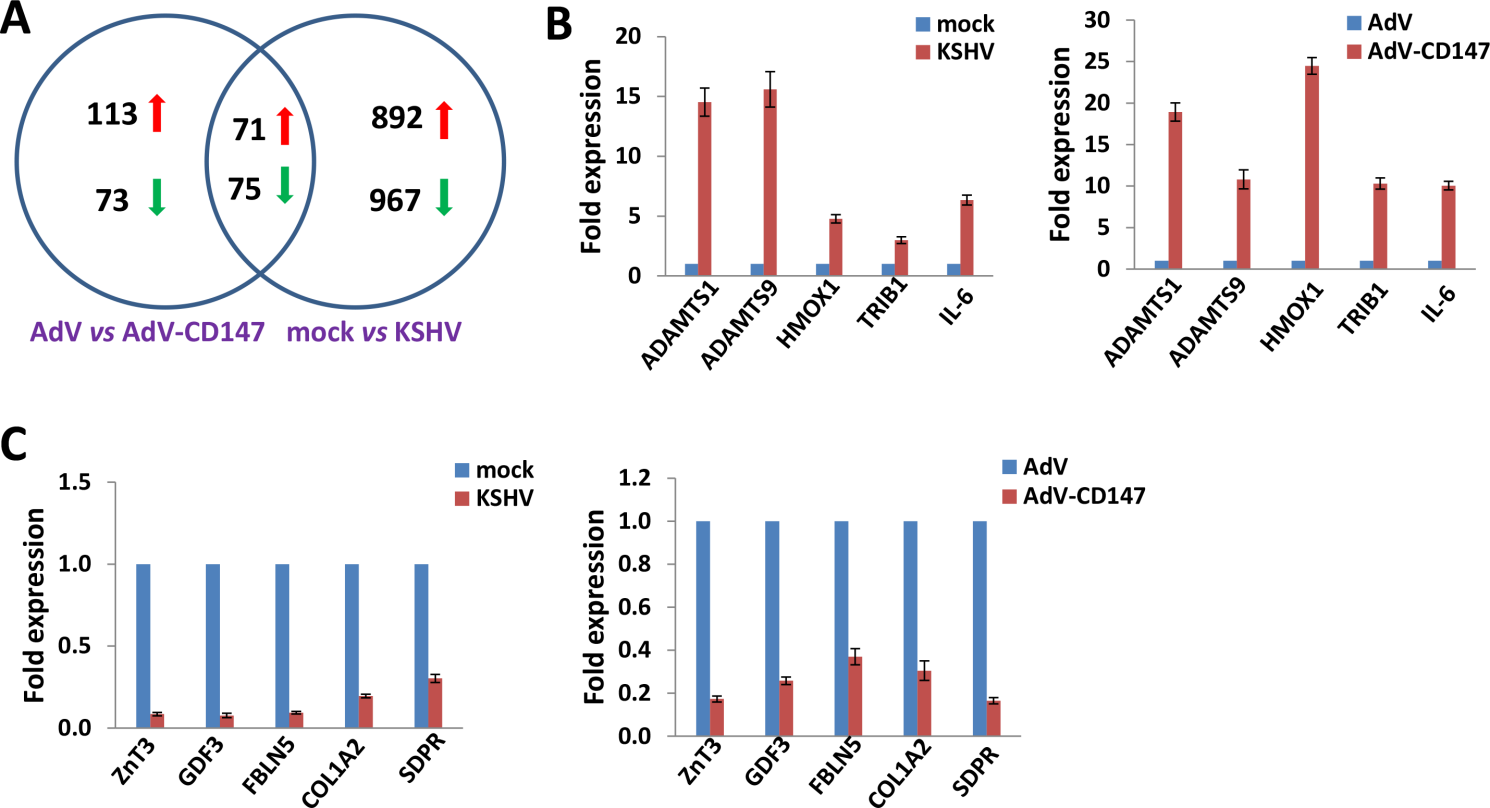
Intersection analysis and experimental validation of gene profile alterations in KSHV-infected and CD147-overexpressed endothelial cells (**A**) The HumanHT-12 v4 Expression BeadChip (Illumina) was used to detect alterations in gene profile in HUVEC cells infected by KSHV (*vs* mock cells), or cells transduced with AdV-CD147 (*vs* AdV-transduced cells). Intersection analysis of significantly altered genes (up/down ≥ 2 fold and *p* < 0.05) was performed using the Illumina GenomeStudio Software. (**B–C**) The transcriptional levels of 5 selected ‘common’ candidate genes that were up-regulated (B) or down-regulated (C) in each set of microarray data were validated by using qRT-PCR. Error bars represent the S.E.M. for 3 independent experiments.

We first used the HumanHT-12 v4 Expression BeadChip (Illumina), which contains more than 47,000 probes derived from the NCBI RefSeq Release 38 and other sources, to study the gene profile altered within CD147-overexpressed HUVEC cells by using a recombinant adenoviral vector AdV-CD147 [11] or within KSHV-infected HUVEC cells. We found that 184 genes were significantly up-regulated and 148 were down-regulated (≥ 2 fold and *p* < 0.05) within CD147-overexpressed endothelial cells; in KSHV-infected cells, 963 genes were up-regulated and 1042 down-regulated. Intersection analysis indicated that 71 “common” genes were significantly up-regulated and 75 were down-regulated in both sets (Figure 1A); the top 10 up-regulated and down-regulated candidate genes were listed in Table 1, respectively. We next selected 5 “common” genes in both sets from Table 1 for validation of their transcriptional change by using qRT-PCR. Our results indicated that all of the 10 selected genes were significantly altered in a manner comparable to those found in the microarray data, demonstrating the credibility of our microarray analysis. Specifically, *ADAMTS1*, *ADAMTS9*, *HMOX1*, *TRIB1* and *IL-6* were significantly up-regulated, while *ZnT3*, *GDF3*, *FBLN5*, *COL1A2*, *SDPR* were significantly down-regulated within either CD147-overexpressed or KSHV-infected endothelial cells (Figure 1B–1C).

**Table 1:** The top 10 ‘common’ candidate genes upregulated or downregulated within both CD147-overexpressed and KSHV-infected HUVEC cells

Gene symbol	Description	Fold change	
		CD147-overexpressed	KSHV-infected
ADAMTS1	A disintegrin and metalloproteinase with thrombospondin motifs 1	16.72	19.26
HMOX1	Heme oxygenase 1	25.8	2.31
RASD1	Dexamethasone-induced Ras-related protein 1	19.74	2.21
ADAMTS9	A disintegrin and metalloproteinase with thrombospondin motifs 9	6.69	12.89
TMEM158	Transmembrane protein 158	14.15	2.44
TRIB1	Tribbles homolog 1	12.23	3.58
DUSP12	Dual specificity protein phosphatase 12	12.8	2.81
IL6	Interleukin-6	8.13	6.69
RGS2	Regulator of G-protein signaling 2	3.73	10.64
CXCL2	C-X-C motif chemokine 2	11.96	2.35
ZnT3	Zinc transporter 3	0.13	0.13
GDF3	Growth/differentiation factor 3	0.16	0.12
MYH10	Myosin-10	0.03	0.26
LTBP2	Latent-transforming growth factor beta-binding protein 2	0.12	0.25
FBLN5	Fibulin-5	0.24	0.15
COL1A2	Collagen alpha-2(I) chain	0.24	0.18
STAT1	Signal transducer and activator of transcription 1-alpha/beta	0.07	0.36
GPR126	G-protein coupled receptor 126	0.2	0.23
GPR124	G-protein coupled receptor 124	0.23	0.28
SDPR	Serum deprivation-response protein	0.08	0.47

Interestingly, some of the top altered candidate genes listed have been reported to be closely associated with KSHV pathogenesis. For example, KSHV infection induces heme oxygenase-1 (HMOX-1 or HO-1), an inducible enzyme responsible for the rate-limiting step in heme catabolism, in infected endothelial cells and/or AIDS-KS tissues [14]. Increased HMOX-1 enzymatic activity *in vitro* has been shown to enhance proliferation of KSHV-infected endothelial cells in the presence of free heme. Fibulin-5 (FBLN5), one of the most down-regulated genes, is greatly decreased in KSHV-infected endothelial cells and/or AIDS-KS tissues, while addition of recombinant Fibulin-5 suppresses VEGF production by KSHV-infected endothelial cells [15]. In contrast, some other candidates have never been reported in KSHV pathogenesis but are thought to be involved in progression of other cancers, such as ADAMTS1 and 9. The ADAMTS family of extracellular metalloproteases, including ADAMTS1 and 9, has been widely implicated in remodeling of the tumor microenvironment during cancer development, growth and progression [16–19]. In particular, elevated ADAMTS1 promotes pro-tumorigenic changes such as increased tumor cell proliferation, decreased apoptosis and altered vascularization [20]. Importantly, it facilitates significant peritumoral remodeling of the extracellular matrix (ECM) microenvironment to promote tumor progression and metastasis. For these reasons we chose ADAMTS1 and 9 for further investigations.

We also performed enrichment analysis of the “common” genes in both sets by using the Pathway map, Gene Ontology (GO) Processes and Process Networks modules from Metacore Software (Thompson Reuters) [21]. Our analysis showed that these genes belong to several major cellular function categories, including cellular immune response to inflammation, blood vessel development, regulation of epithelial-mesenchymal transition (EMT), cell adhesion and cell cycle/proliferation (Supplementary Figure S1A–S1C), some of which have been reported closely related to KSHV pathogenesis or tumorigenesis [22, 23]. In addition, the top 2 scored pathway maps and protein networks for these “common” genes were shown in Supplementary Figure S2 and Figure S3, respectively.

### Clinical relevance of CD147 and downstream ADAMTSs within AIDS-KS tissues

**Figure 2:**
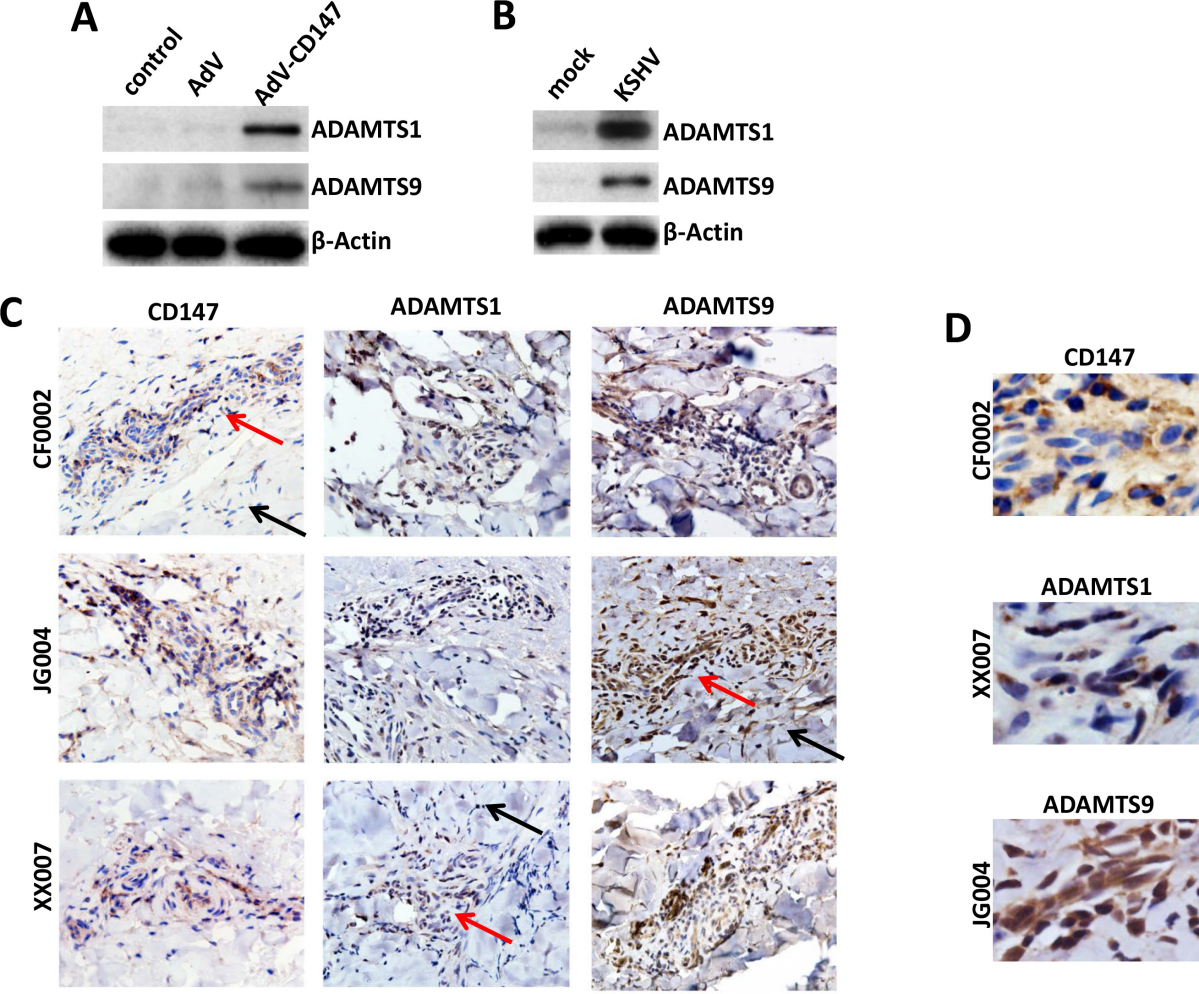
Up-regulation of ADAMTS1 and 9 expression in CD147-overexpressed or KSHV-infected endothelial cells and in AIDS-KS tissues (**A–B**) HUVEC were transduced using a recombinant human CD147-encoding adenovirus (AdV-CD147), or control adenovirus (AdV) for 48 h, or infected by purified KSHV (MOI ∼ 10) for 48 h. Protein expression was measured by immunoblots. (**C–D**) The expression of CD147, ADAMTS1 and ADAMTS9 within KS tumor tissues from 3 cohort HIV+ patients was detected by immunohistochemistry (400x magnification, and tumor cells in selected regions were magnified at 600x in panel D). Red arrows indicate the KS tumor area and black arrows indicate the adjacent area from the same patient.

Due to the important role of ADAMTS family members in cancer progression, we further examined their regulation by CD147 in endothelial cells and their expression in AIDS-KS tumors. We previously demonstrated up-regulation of CD147 expression in HUVEC by either a recombinant CD147 adenovirus (AdV-CD147) transduction or KSHV *de novo* infection [10, 11]. Here we showed the elevated expression of ADAMTS1 and 9 after both treatments in cultured HUVEC cells (Figure 2A–2B). Furthermore, we measured their expression levels in KS tumor tissues directly isolated from cohort 3 AIDS patients without chemotherapy treatments. Our results showed the strong expression of CD147, as well as ADAMTS1 and 9 within KS tumor tissues from all the patients (CF0002, JG004 and XX007) (Figure 2C). In addition, we found that CD147, ADAMTS1 and 9 were all expressed mostly in “spindle cells”, the typical LANA+ KS tumor cells [24] (Figure 2D). Taken together, these data strongly suggest the involvement of CD147 and downstream ADAMTS proteins in KSHV-related malignancies, specifically KS.

### Targeting CD147 and downstream ADAMTSs represses pro-inflammatory, pro-angiogenic cytokine production and endothelial cell invasion

**Figure 3:**
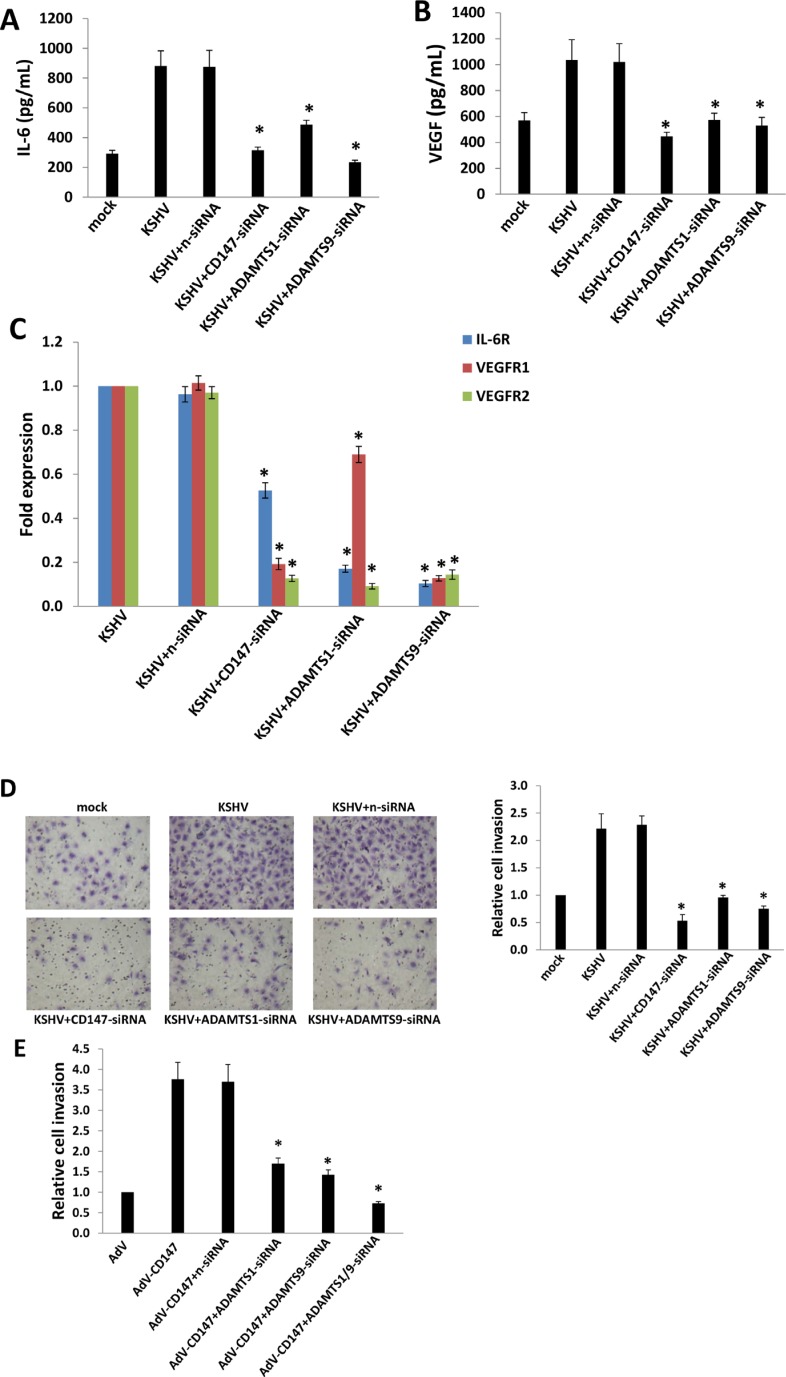
Targeting CD147 and downstream ADAMTSs represses expression of IL-6, VEGF, their respective receptors, and KSHV-infected endothelial cell invasion (**A–B**) HUVEC were incubated with media (mock) or purified KSHV for 2 h, with or without prior transfection for 48 h with negative control siRNA (n-siRNA), *CD147*-siRNA, *ADAMTS1*-siRNA or *ADAMTS9*-siRNA, respectively. Cells were subsequently incubated in fresh media for an additional 24 h. The concentrations of IL-6 and VEGF in culture supernatants were determined using ELISAs. (**C–D**) HUVEC were treated as above, then the expression of cytokine receptor genes and cell invasiveness were measured by qRT-PCR and transwell assays, respectively. (**E**) HUVEC were transfected for 48 h with negative n-siRNA, *ADAMTS1*-siRNA or *ADAMTS9*-siRNA, respectively, then transduced with AdV-CD147 or control AdV for 48 h. Cell invasion was assessed by transwell assays. Error bars represent the S.E.M. for 3 independent experiments. * = *p* < 0.05.

Pro-inflammatory and pro-angiogenic cytokines, in particular IL-6 and VEGF, are secreted by KSHV-infected cells. Their presence within KS lesions and the peripheral circulation of KS patients is thought to facilitate KSHV-associated cellular pathogenesis and angiogenesis [25–27]. Moreover, acquisition of a migratory or invasive phenotype represents one hallmark of KSHV-infected endothelial cells, with implications for both viral dissemination and angiogenesis within KS lesions [28]. Here our results indicated that targeting *CD147*, *ADAMTS1* or *9* by RNAi significantly reduced IL-6 and VEGF production from KSHV-infected HUVEC, as well as the transcripts for their receptors (IL-6R, VEGFR1 and VEGFR2) (Figure 3A–3C and Supplementary Figure S4). By using the transwell assays, we found that silencing of *CD147*, *ADAMTS1* or *9* effectively reduced KSHV-infected HUVEC invasion (Figure 3D). Moreover, silencing of *CD147*, *ADAMTS1* or *9* also significantly reduced CD147-overexpressed HUVEC invasion (Figure 3E), demonstrating that these ADAMTSs are indeed required for CD147-mediated endothelial cell invasion.

### Establishment of a KS-like nude mouse model using TIVE-LT cells

**Figure 4:**
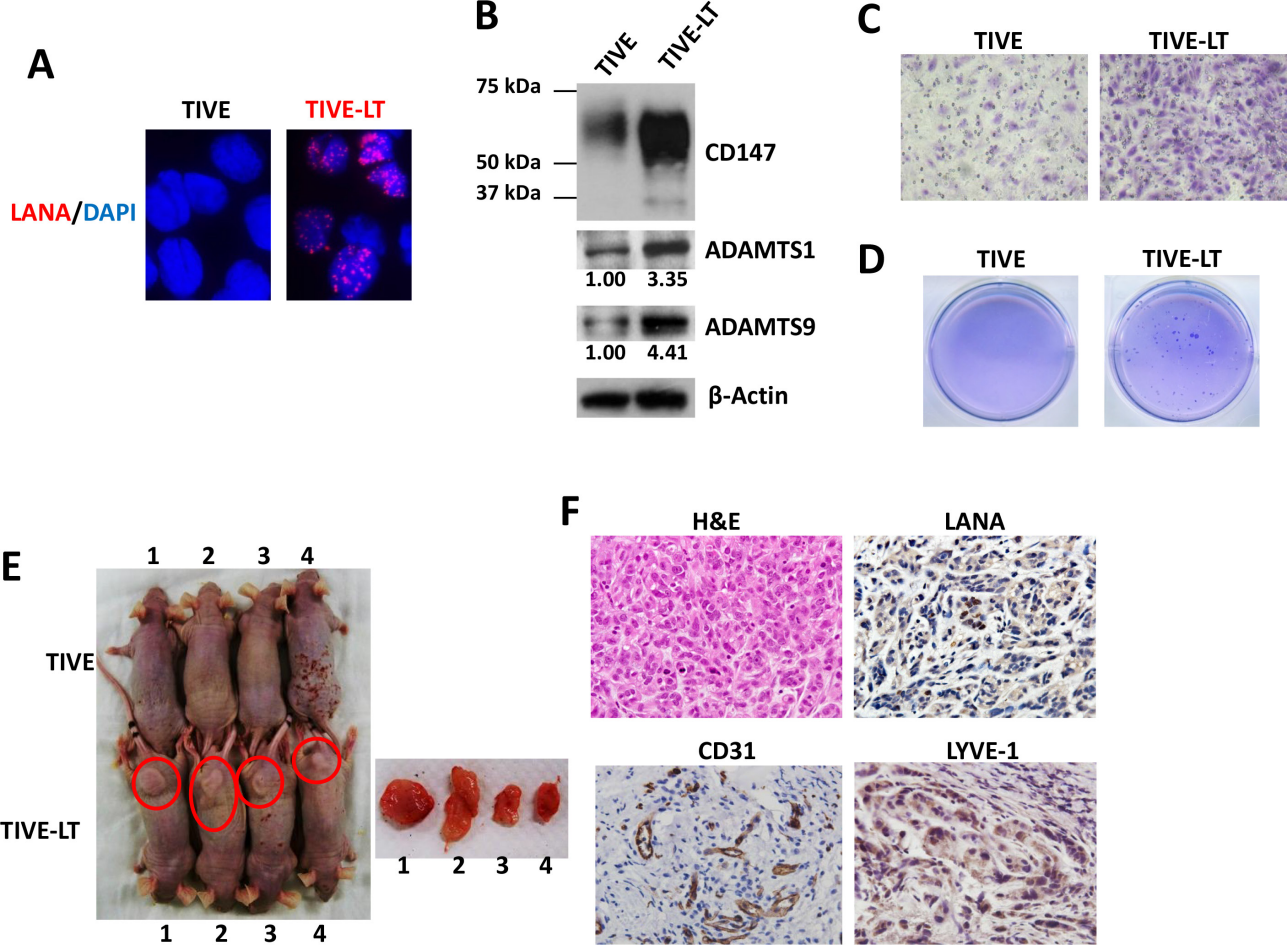
Establishment of a KS-like nude mouse model using TIVE-LT cells (**A**) IFA was used to detect nuclear LANA expression in TIVE-LT cells, and TIVE cells as a negative control. (**B–D**) Protein expression (B), cell invasion (C) and anchorage-independent growth (D) were measured by immunoblots, transwell and soft agar assays, respectively. (**E**) TIVE or TIVE-LT cells (approximately 5 × 10^5^ cells were mixed at a ratio of 1:1 with growth factor-depleted Matrigel) were injected subcutaneously into nude mice. Mice injected with TIVE-LT at 28d developed tumors (4/4) as shown by red circle, while none (0/4) of the mice injected with TIVE cells did. (**F**) H & E staining of representative tumor tissues isolated from TIVE-LT-injected mice displayed KS-like features, including a mixture of elongated-spindle-cell and undifferentiated morphologies with prominent mitotic figures. Immunohistochemistry (IHC) staining showed that most tumor cells expressed KS-specific viral/cellular marker molecules.

KSHV-infected primary endothelial cells usually cannot form tumors even in immunodeficiency mice [24]. Recently, a stable KSHV latency cell-line, named TIVE-LT cells has been established, which can induce KS-like tumor formation in nude mice [13]. Here we found robust LANA expression in the nuclei of TIVE-LT cells (a molecular marker for KSHV latency) [29], while none was observed in the uninfected parental TIVE cells (Figure 4A). Interestingly, TIVE-LT cells displayed much higher levels of CD147, ADAMTS1 and 9 expression than TIVE cells (Figure 4B). Accordingly, TIVE-LT cells possessed much stronger capacities for cell invasion and anchorage-independent growth than TIVE cells, the latter forming almost no colonies in the soft agar assay (Figure 4C–4D).

We next examined the ability of TIVE and TIVE-LT cells to form tumors after subcutaneous injection into nude mice. We found that all the mice injected with TIVE-LT cells developed visible tumors by 28 day (4/4), while none (0/4) of the mice injected with TIVE cells did (Figure 4E). H & E staining of tumor tissues from TIVE-LT-injected mouse displayed KS-like features, including a mixture of elongated-spindle-cell and undifferentiated morphologies with prominent mitotic Figures. Immunohistochemistry (IHC) staining showed that most tumor cells expressed KS-specific viral/cellular marker molecules [24], such as LANA, CD31 and LYVE-1 (Figure 4F).

### Targeting CD147 and downstream ADAMTS1 suppresses KSHV+ TIVE-LT cell tumorigenesis *in vivo*

**Figure 5:**
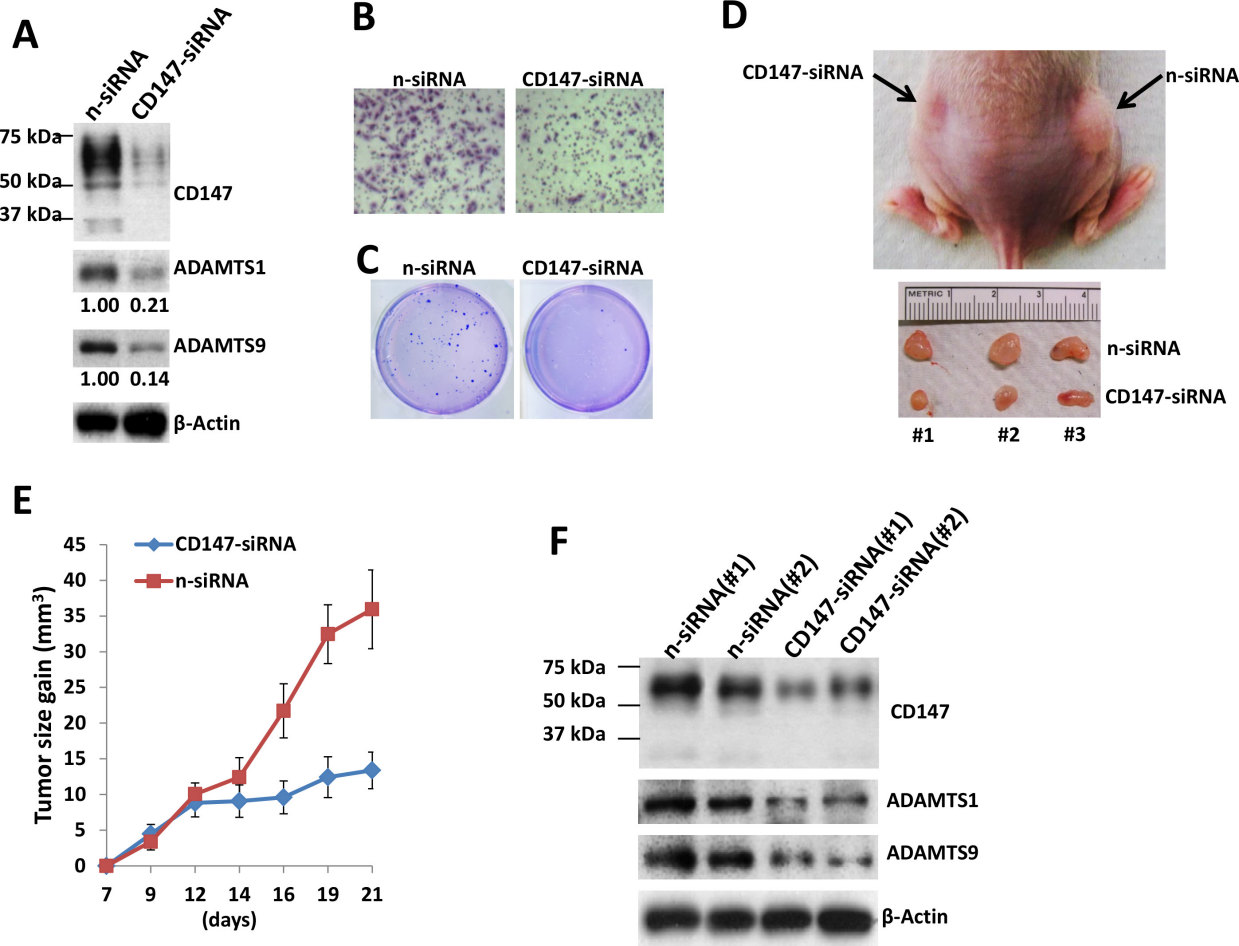
Targeting CD147 significantly suppresses TIVE-LT cell tumorigenesis *in vivo* (**A–C**) TIVE-LT cells were transfected with negative control siRNA (n-siRNA) or CD147-siRNA for 48 h, then protein expression (A), cell invasion (B) and anchorage-independent growth (C) were measured by immunoblots, transwell and soft agar assays, respectively. (**D–E**) TIVE-LT cells transfected with n-siRNA or CD147-siRNA (approximately 5 × 10^5^ cells were mixed at a ratio of 1:1 with growth factor-depleted Matrigel) were injected subcutaneously into the right and left flanks of nude mice (3 mice per group), respectively. The mice were observed and measured every 2∼3 d for the presence of palpable tumors for 21 d. (**F**) Protein expression was measured by immunoblots of tumor lysates from 2 TIVE-LT/n-siRNA or TIVE-LT/CD147-siRNA injected flanks of mice sacrificed at day 21, respectively.

Since TIVE-LT cells displaying high CD147 expression, we found that silencing of CD147 by RNAi reduced the expression of ADAMTS1 and 9 (Figure 5A), as well as their transcripts (data not shown). Silencing of CD147 by RNAi significantly blocked TIVE-LT cell invasion and anchorage-independent growth, when compared to negative control siRNA (n-siRNA) group (Figure 5B–5C). We also observed long-term “knock-down” of CD147 expression by RNAi for at least 2 weeks *in vitro* culture (Supplementary Figure S5). Next, we injected TIVE-LT cells transfected with n-siRNA or *CD147*-siRNA subcutaneously into the right and left flanks of nude mice, respectively. These mice were observed and measured every 2∼3 day for the presence of palpable tumors for 21 days. Our results indicated that silencing of CD147 significantly repressed tumor growth in nude mice. Mice injected with *CD147*-siRNA formed smaller tumors when compared to n-siRNA groups at 21 days (Figure 5D–5E). Immunoblot results confirmed the higher levels of CD147, ADAMTS1 and 9 expression in tumor lysates from mice with n-siRNA than those from mice with *CD147*-siRNA, demonstrating the successful silencing of CD147 and downstream ADAMTSs *in vivo* (Figure 5F). Our additional data indicated that direct silencing of ADAMTS1 by siRNA also significantly repressed tumor growth in nude mice (Supplementary Figure S6). Taken together, these data support the important role of CD147, ADAMTS1 and 9 as cellular co-factors for KSHV-related tumorigenicity and KS development.

## DISCUSSION

Our previous studies have demonstrated that CD147 is an important contributor to KSHV-induced endothelial cell invasiveness, through a complex of underlying mechanisms [10, 11, 30]. However to our knowledge, there are no current data exploring the global gene profile controlled by CD147 in primary endothelial cells, in particular KSHV-infected cells, which are the major cellular components of KS tissues. In the current study, we identified at least ∼150 genes potentially controlled by CD147 in KSHV-infected endothelial cells by using Illumina microarray. Interestingly, only a few of them have been reported associated with KSHV pathogenesis, e.g. HMOX-1 and FBLN5 [14, 15], while most of them remain functionally unknown with respect to KSHV-related diseases. The enrichment analysis indicates that these genes belong to several major cellular function categories closely related to KSHV pathogenesis, such as EMT and blood vessel development [22, 23]. Although the role of EMT in KS development remains unclear, another similar pathophysiologic behavior, called as endothelial-to-mesenchymal transformation (EndMT), has been described during KSHV infection. Cheng *et al.* reported that KSHV-induced EndMT was initiated by the viral FLICE inhibitory protein (vFLIP) or vGPCR through Notch pathway activation, leading to cell invasiveness dependent upon membrane-type-1 matrix metalloproteinase (MT1-MMP) [31]. Gasperini *et al.* reported that canonical Notch signaling, including Slug and ZEB1, was required for KSHV-induced EndMT which increased invasiveness and survival in infected endothelial cells [32]. Interestingly, CD147 has been found involved in EMT and closely associated phenomena in cancer cells [33–35]. Additionally, the expression of functional CD147 and MMP-2 was significantly decreased in Notch1-deficient breast cancer cells which displayed impaired migration and invasion [36].

Here we showed that two specific metalloproteases, ADAMTS1 and 9 were up-regulated by CD147 and contributed to KSHV-induced cell invasiveness. Previous reports showed that CD147 induced production of several MMPs [7–9] and urokinase-type plasminogen activator [37], but to our knowledge this is the first report showing the up-regulation of ADAMTSs by CD147. Moreover, CD147, ADAMTS1 and 9 were strongly expressed within AIDS-KS tumor tissues, demonstrating their potential clinical relevance as well as biomarker or therapeutic values. However, it remains unknown how CD147 can increase the expression and maturation of ADAMTS1 and 9, which may require a variety of additional factors. For example, ADAMTS1 is synthesized as a pro-zymogen and undergoes N-linked glycosylation following protein translation [38]. The secretion of ADAMTS1 to the ECM requires the excision of its pro-domain from the 87kD mature protein by furin-related endopeptidases [38]. Interestingly, ADAMTS1 can be transiently induced by hypoxia in endothelial cells, which is mediated by hypoxia-inducible factor 1 (HIF-1) binding to its promoter region [39]. Moreover, CD147 can be induced in a similar manner in various carcinoma cells [40]. It has been reported that several KSHV-encoded proteins such as LANA, vIRF-3 or vGPCR can activate HIF-1 and contribute to angiogenesis, tumorigenesis or virus replication [41–44]. Therefore, we are interested to understand whether these viral proteins (latent or lytic) are able to up-regulate the expression of ADAMTS1 and 9 and the underlying mechanisms.

Our data showed that silencing of CD147 by RNAi significantly repressed tumor growth in a nude mouse model, and that these treated tumor tissues displayed less blood vessel formation and less tumor cells when compared to n-siRNA groups (data not shown). We assume this is partially due to impaired ECM dynamics in the tumor microenvironment through down-regulation of ADAMTS1 and 9. Our recent study indicated that silencing of CD147 by RNAi greatly reduced the production of hyaluronan, an important ECM component, through down-regulation of the hyaluronan synthase gene 1 (*Has1*) within KSHV-infected HUVEC cells [30]. Interestingly, we found that silencing of CD147 also significantly reduced hyaluronan production from TIVE-LT cells, in this case through down-regulation of *Has2* and *Has3* instead of *Has1* (Dai *et al.*, unpublished data). Therefore, we will evaluate the strategies targeting ECM formation and/or components as potential therapies against KS development in future study.

## MATERIALS AND METHODS

### Cell culture, reagents and infection protocol

Body cavity-based lymphoma cells (BCBL-1, KSHV^+^/EBV^−^) were maintained in RPMI 1640 medium (Gibco) with supplements as described previously [45]. Telomerase-immortalized human umbilical vein endothelial (TIVE) and KSHV long-term-infected TIVE (TIVE-LT) cells were cultured as previously described [13]. Human umbilical vein endothelial cells (HUVEC) were grown in DMEM/F-12 50/50 medium (Cellgro) supplemented with 5% FBS. All cells were incubated at 37°C in 5% CO_2_. All experiments were carried out using cells harvested at low (< 20) passages. To obtain KSHV for infection experiments, BCBL-1 cells were incubated with 0.6 mM valproic acid for 6 days, and purified virus was concentrated from culture supernatants and infectious titers were determined as described previously [46]. For overexpression of CD147, HUVEC were transduced as previously described with a recombinant adenoviral vector (MOI ∼ 10) encoding CD147 (AdV-CD147), or a control vector (AdV), for 48 h prior to subsequent analyses [11].

### Microarray

Total RNA was isolated using Qiagen RNeasy kit (Qiagen), and 500 ng of total RNA was used to synthesize dscDNA. Biotin-labeled RNA was generated using the TargetAmp-Nano Labeling Kit for Illumina Expression BeadChip (Epicentre), according to the manufacturers' instructions, and hybridized to the HumanHT-12 v4 Expression BeadChip (Illumina), which contains more than 47,000 probes derived from the NCBI RefSeq Release 38 and other sources, at 58°C for 16 h. The chip was washed, stained with streptavadin-Cy3, and scanned with the Illumina BeadStation 500 and BeadScan. Using the Illumina's GenomeStudio software, we normalized the signals using the “cubic spline algorithm” that assumes that the distribution of transcript abundance is similar in all samples, according to the method proposed by Workman *et al.* [47]. The background signal was removed using the “detection *p*-value algorithm” to remove targets with signal intensities equal or lower than that of irrelevant probes (with no known targets in the human genome but thermodynamically similar to the relevant probes). The microarray experiments were performed twice for each group and the average values were used for analysis. Common and unique sets of genes and enrichment analysis were performed using the MetaCore Software (Thompson Reuters) as previously reported [21]. The microarray original data have been submitted to Gene Expression Omnibus (GEO) database (Accession number: GSE69067).

### RNA interference

*CD147*, *ADAMTS1* or *ADAMTS9* ON-TARGET plus SMART pool siRNA, or negative control siRNA (n-siRNA) (Dharmacon), were delivered using the DharmaFECT transfection reagent according to the manufacturer's instructions.

### Immunoblotting

Total cell lysates (20 μg) were resolved by 10% SDS–PAGE, transferred to nitrocellulose membranes, and immunoblotted with antibodies for CD147 (BD), ADAMTS1, ADAMTS9 (Cell Signaling) and β-Actin (Sigma) for loading controls. Immunoreactive bands were identified using an enhanced chemiluminescence reaction (Perkin-Elmer), visualized by autoradiography and quantitated using Image-J software.

### Immunofluorescence

Cells were incubated in 1:1 methanol-acetone at −20°C for fixation and permeabilization, then with a blocking reagent (10% normal goat serum, 3% bovine serum albumin, and 1% glycine) for an additional 30 minutes. Cells were then incubated for 1 h at 25°C with 1:1000 dilution of a rat anti-LANA monoclonal antibody (ABI) followed by 1:200 dilution of a goat anti-rat secondary antibody conjugated to Texas Red (Invitrogen). For identification of nuclei, cells were subsequently counterstained with 0.5 mg/mL 4′,6-diamidino-2-phenylindole (DAPI; Sigma) in 180 mM Tris-HCl (pH 7.5). Slides were washed once in 180 mM Tris-HCl for 15 minutes and prepared for visualization using a Leica TCPS SP5 AOBS confocal microscope.

### qRT-PCR

Total RNA was isolated using the RNeasy Mini kit (QIAGEN), and cDNA was synthesized from equivalent total RNA using a SuperScript III First-Strand Synthesis SuperMix Kit (Invitrogen) according to the manufacturer's instructions. Primers used for amplification of target genes are displayed in Supplementary Table 1. Amplification was carried out using an iCycler IQ Real-Time PCR Detection System, and cycle threshold (Ct) values were tabulated in duplicate for each gene of interest in each experiment. “No template” (water) controls were used to ensure minimal background contamination. Using mean Ct values tabulated for each gene, and paired Ct values for β-*actin* as a loading control, fold changes for experimental groups relative to assigned controls were calculated using automated iQ5 2.0 software (Bio-rad).

### ELISA

Concentrations of IL-6 and VEGF in culture supernatants were determined using human IL-6 (eBioscience) and VEGF-A (Pierce Biotechnology) ELISA kits according to the manufacturers' instructions.

### Transwell invasion assays

Matrigel Invasion Chambers (BD) were hydrated for 4 h at 37°C with culture media. Following hydration, media in the bottom of the well was replaced with fresh media, then 2 × 10^4^ HUVEC, TIVE or TIVE-LT cells were plated in the top of the chamber. After 24 h, cells were fixed with 4% formaldehyde for 15 min at room temperature and chambers rinsed in PBS prior to staining with 0.2% crystal violet for 10 min. After washing the chambers, cells at the top of the membrane were removed and cells at the bottom of the membrane counted using a phase contrast microscope. Relative invasion was determined for cells in experimental groups as follows: relative invasion = # invading cells in experimental group / # invading cells in control groups.

### Soft agar assays

A base layer containing 0.5% agarose medium and 5% FCS was poured into six-well plates. Then, 10,000 cells were mixed with 0.4% agarose in Earl's minimal essential medium (EMEM) containing 5% FCS to form a single-cell suspension. After being seeded, the plates were incubated for 2 weeks. Colonies were stained with 0.005% crystal violet and photographed under a phase-contrast microscope (Leica DFC320).

### KS-like nude mouse model

Cells were counted and washed once in ice-cold PBS, and 5 × 10^5^ cells in 50 μL PBS plus 50 μL growth factor-depleted Matrigel (BD Biosciences) were injected subcutaneously into the flanks of nude mice (Jackson Laboratory). The mice were observed and measured every 2∼3 d for the presence of palpable tumors. At the end of experiment, the tumors were excised from the site of injection for subsequent analysis such as immunoblots and immunohistochemistry. All protocols were approved by the LSUHSC Animal Care and Use Committee in accordance with national guidelines.

### KS tumor tissues from HIV^+^ patients and immunohistochemistry

KS tissues from HIV-infected patients were provided by the LSUHSC HIV Outpatient (HOP) Clinic and Biospecimens Bank (LSUHSC IRB approved No. 8079). Formalin-fixed, paraffin-embedded tissues were microtome-sectioned to a thickness of 4 uM, placed on electromagnetically charged slides (Fisher Scientific), and stained with hematoxylin & eosin (H & E) for routine histologic analysis. Immunohistochemistry was performed using the Avidin-Biotin-Peroxidase complex system, according to the manufacturer's instructions (Vectastain Elite ABC Peroxidase Kit; Vector Laboratories). In our modified protocol, sections were deparaffinized in xylene and re-hydrated through a descending alcohol gradient. For non-enzymatic antigen retrieval, slides were heated in 0.01 M sodium citrate buffer (pH 6.0) to 95°C under vacuum for 40 min and allowed to cool for 30 min at room temperature, then rinsed with PBS and incubated in MeOH/3% H_2_O_2_ for 20 min to quench endogenous peroxidase. Slides were then washed with PBS and blocked with 5% normal goat serum in 0.1% PBS/BSA for 2 h at room temperature, then incubated overnight with indicated antibody at 1:200-1:400 dilution in 0.1% PBS/BSA. The following day, slides were incubated with appropriate secondary antibody at room temperature for 1 h, followed by avidin-biotin peroxidase complexes for 1 h at room temperature. Finally, slides were developed using a diaminobenzidine substrate, counterstained with hematoxylin, dehydrated through an ascending alcohol gradient, cleared in xylene, and coverslipped with Permount. Images were collected using an Olympus BX61 microscope equipped with a high resolution DP72 camera and CellSense image capture software.

### Statistical analysis

Significance for differences between experimental and control groups was determined using the two-tailed Student's *t*-test (Excel 8.0), and *p* values < 0.05 were considered significant.

## SUPPLEMENTARY MATERIALS FIGURES AND TABLE


