# Supplementary material for: CD147 and downstream ADAMTSs promote the tumorigenicity of Kaposi's sarcoma-associated herpesvirus infected endothelial cells
Source: Oncotarget. 2015 Dec 12;7(4):3806–18. doi: 10.18632/oncotarget.6584 (PMC4826171; doi:10.18632/oncotarget.6584)
Supplement: Supplementary file 1 [file oncotarget-07-3806-s001.pdf]

CD147 and downstream ADAMTSs promote the tumorigenicity of Kaposi's sarcoma-associated herpesvirus infected endothelial cells

Supplementary Materials

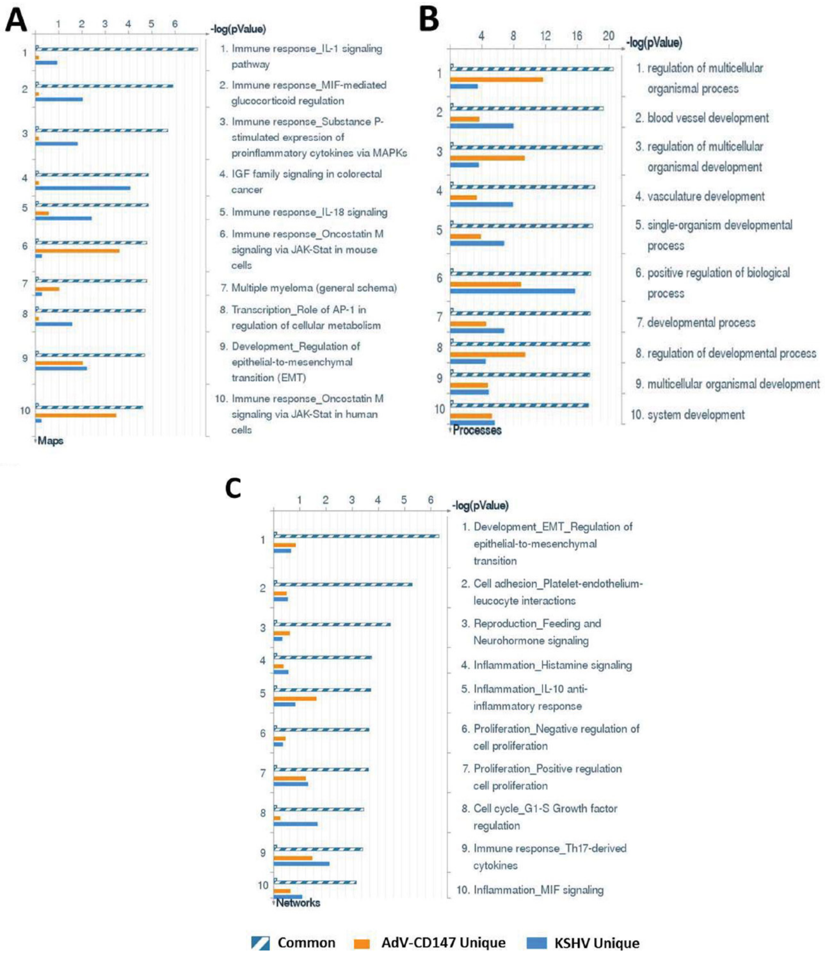

Supplementary Figure S1: The enrichment analysis of gene profile alterations in KSHV-infected and CD147-overexpressed endothelial cells. (**A–C**) The HumanHT-12 v4 Expression BeadChip (Illumina) was used to detect alterations in gene profile in HUVEC cells infected by KSHV (*vs* mock cells), or cells transduced with AdV-CD147 (*vs* AdV-transduced cells). The enrichment analysis of gene profiles significantly altered(up/down ≥ 2fold and $p < 0.05$) in KSHV-infected and CD147-overexpressed HUVEC cells was performed using the Metacore Software (Thompson Reuters) Modules: Pathway Maps (A), Gene Ontology Processes (B) and Process Networks (C).

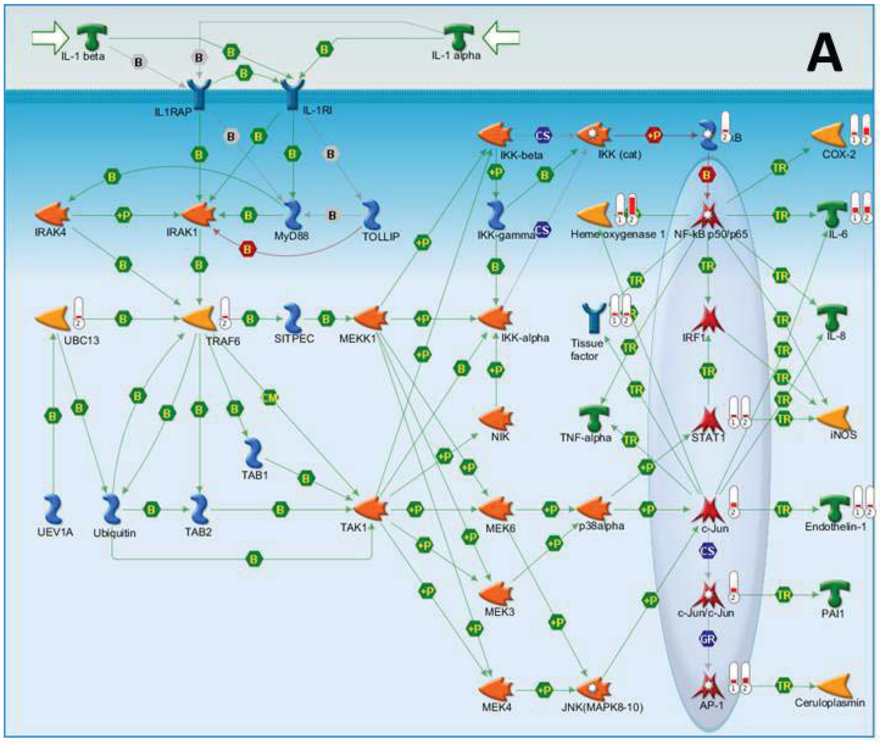

Immune response IL-1 signaling pathway

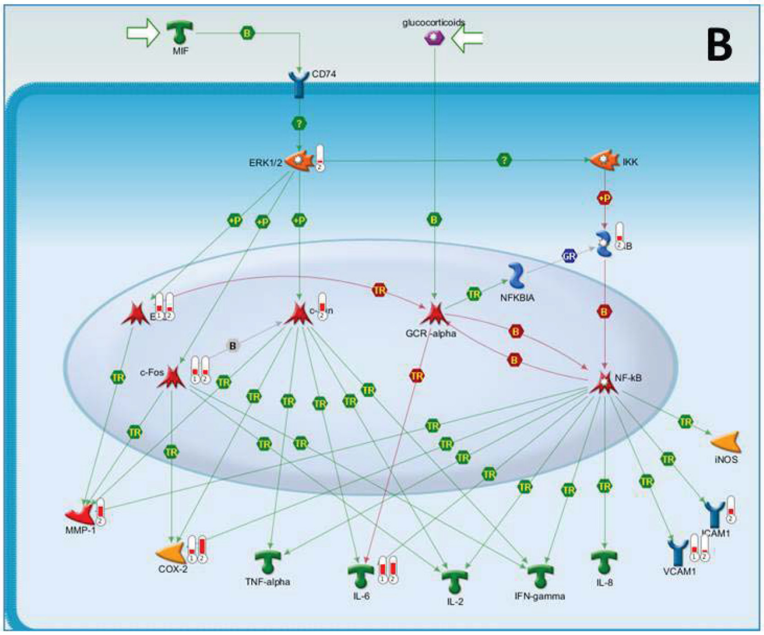

Immune response MIF mediated glucocorticoid regulation

Supplementary Figure S2: The top 2 scored maps (maps with the lowest *p*-value) based on the enrichment distribution sorted by 'common' gene set altered in KSHV-infected and CD147-overexpressed endothelial cells. (**A–B**) Experimental data from all files is linked to and visualized on the maps as thermometer like figures. Up-ward thermometers have red color and indicate upregulated signals and down ward (blue) ones indicate downregulated expression levels of the genes.

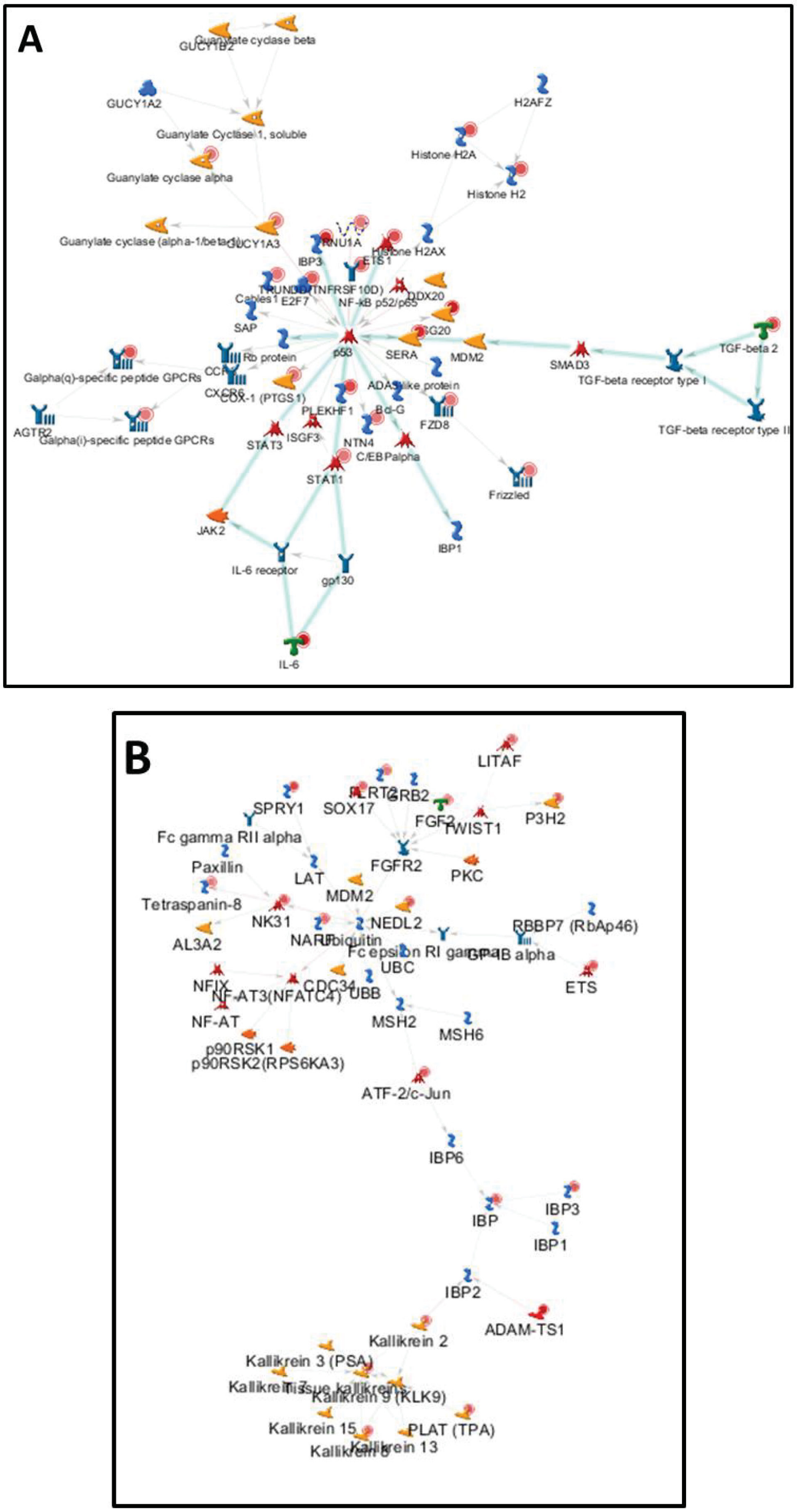

Supplementary Figure S3: The top 2 scored (by the number of pathways) AN networks from 'common' gene sets altered within KSHV-infected and CD147-overexpressed endothelial cells. (**A–B**) Thick cyan lines indicate the fragments of canonical pathways. Upregulated genes are marked with red circles; downregulated with blue circles. The **'**checkerboard**'** color indicates mixed expression for the gene between files or between multiple tags for the same gene. Data was produced by the Metacore Software (Thompson Reuters).

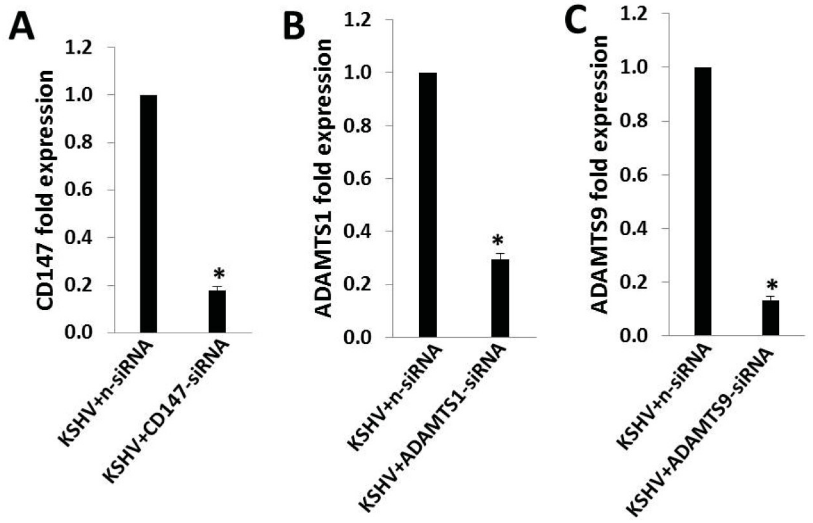

Supplementary Figure S4: Effective silencing of CD147 and downstream ADAMTSs by RNAi in KSHV-infected endothelial cells. (**A–C**) HUVEC were transfected with negative control siRNA (n-siRNA), *CD147*-siRNA, *ADAMTS1*-siRNA or *ADAMTS9*-siRNA, respectively, then infected by purified KSHV. Gene transcripts were quantified by qRT-PCR. Error bars represent the S.E.M. for 3 independent experiments. * = $p < 0.05$.

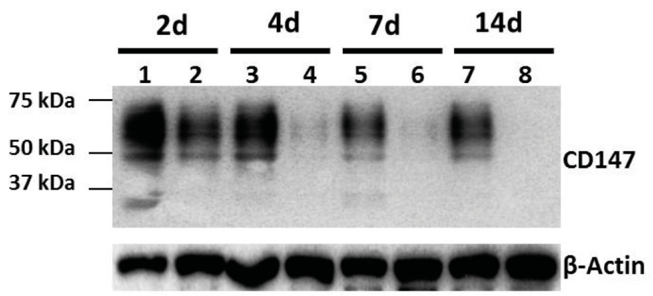

Supplementary Figure S5: Effective long-term silencing of CD147 by RNAi in TIVE-LT cells *in vitro*. TIVE-LT cells were transfected with negative control siRNA (n-siRNA) or *CD147*-siRNA, then cell lysates were 3 collected at indicated time and protein expression was measured by immunoblots. Lane 1, 3, 5, 7: TIVE-LT + n-siRNA; lane 2, 4, 6, 8: TIVE-LT + *CD147*-siRNA.

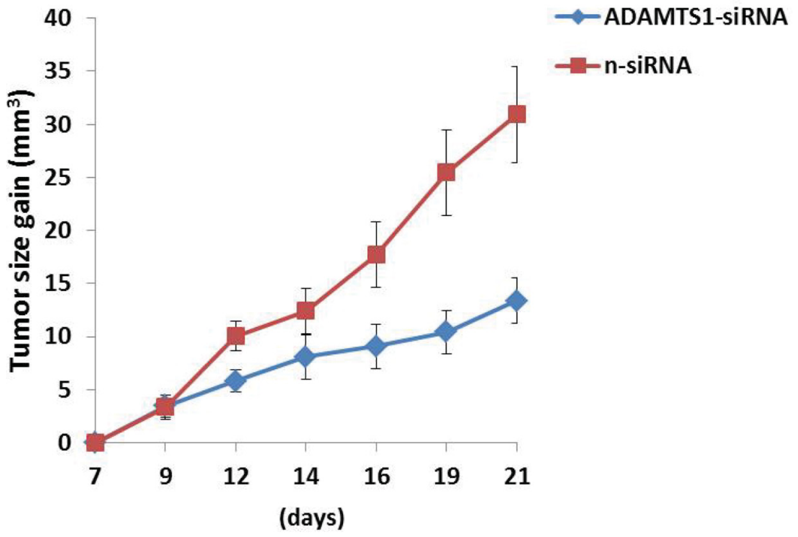

Supplementary Figure S6: Targeting ADAMTS1 significantly suppresses TIVE-LT cell tumorigenesis *in vivo*. TIVE-LT cells transfected with n-siRNA or *ADAMTS1*-siRNA (approximately 5×10^5 cells were mixed at a ratio of 1:1 with growth factor-depleted Matrigel) were injected subcutaneously into the right and left flanks of nude mice (3 mice per group), respectively. The mice were observed and measured every 2~3 d for the presence of palpable tumors for 21 d.

Supplementary Table S1: Primer sequences for qRT-PCR in this study

Gene	Sequences (5′→ 3′)
ADAMTS1	*sense AGCCTCAGAATCCCATACA*<br>*antisense TTGCCGTTGATACACCAT*
ADAMTS9	*sense CCAGCAGGAGTGAATGTAG*<br>*antisense AGTCCAGGCAGAATAGCG*
HMOX1	*sense TTTGAGGAGTTGCAGGAGC*<br>*antisense AGGACCCATCGGAGAAGC*
TRIB1	*sense GCCACCAGTCAGCCATCGT*<br>*antisense GGGTATCGTCCAACCAGAA*
IL-6	*sense GTCCAGTTGCCTTCTCCC*<br>*antisense GCCTCTTTGCTGCTTTCA*
ZnT3	*sense GCCCTTCCACCACTGCCACA*<br>*antisense TGCCAGATACCCGCCGACCA*
GDF3	*sense AAGAAAGGGAACAGTTGACA*<br>*antisense CCAGGAATAACCCGAAAT*
FBLN5	*sense CAACACCTACGGCTCTTT*<br>*antisense TCAGATAAGGCTCCTCACA*
COL1A2	*sense GTGGTGGTTATGACTTTGGTT*<br>*antisense GCGAGCTGGGTTCTTTCTA*
SDPR	*sense CTCCGACGCAACCATTTC*<br>*antisense GCTTTCTTGAGGCTATCCACT*
IL-6R	*sense AGTTCCAGCTTCGATACCGAC*<br>*antisense GTATTGTCAGACCCAGAGCTG*
VEGFR1	*sense GACTAGATAGCGTCACCAG*<br>*antisense AATACTCCGTAAGACCACA*
VEGFR2	*sense AAAGGGTGGAGGTGACTG*<br>*antisense GACATAAATGACCGAGGC*
β-actin	*sense GGAAATCGTGCGTGACATT*<br>*antisense GACTCGTCATACTCCTGCTTG*
